# Seatbelt Injury Causing Small Bowel Devascularisation: Case Series and Review of the Literature

**DOI:** 10.1155/2011/675341

**Published:** 2011-04-07

**Authors:** Vincent O'Dowd, Christine Kiernan, Aoife Lowery, Waqar Khan, Kevin Barry

**Affiliations:** Department of Surgery, Mayo General Hospital, Castlebar, Mayo, Ireland

## Abstract

The use of seatbelts has increased significantly in the last twenty years, leading to a decrease in mortality from road traffic accidents (RTA). However, this increase in seatbelt use has also led to a change in the spectrum of injuries from RTA; abdominal injuries, particularly intestinal injuries have dramatically increased with the routine use of seatbelts. Such intestinal injuries frequently result from improper placement of the “lap belt”. We present 3 cases in which passengers wearing a seatbelt sustained significant devascularisation injuries to the small bowel requiring emergency surgical intervention. A high index of suspicion is crucial in such cases to prevent delays in diagnosis that can lead to severe complications and adverse outcomes. It is evident that while advocating seatbelt use, the importance of education in correct seatbelt placement should also be a focus of public health strategies to reduce RTA morbidity and mortality.

## 1. Introduction

The mortality of passengers in road traffic accidents (RTA) is decreasing as a result of increased use of seatbelts; according to a survey by the National Roads Authority (NRA) in Ireland, compliance with seatbelt wearing has increased from 53% in 1991 to 86% in 2005 [1]. However, the use of seatbelts is associated with a unique injury profile collectively termed “the seatbelt syndrome” which includes injuries to the intestinal viscera, tears and perforations of the gastrointestinal tract and its mesentery and lumbar fracture dislocations [2]. These injuries are more prevalent in the paediatric population due to ill-fitting lap belts [3]. However, they also occur in adults, particularly when a seatbelt is worn incorrectly due to suboptimal placement, inadequate securing or patient factors such as obesity and poor positioning/slouching. We report 3 cases from Mayo General Hospital which demonstrate significant intra-abdominal/mesenteric injury sustained through seatbelt use in RTA.

## 2. Cases

The three cases are summarized in Table 1. 

**Figure 1:**
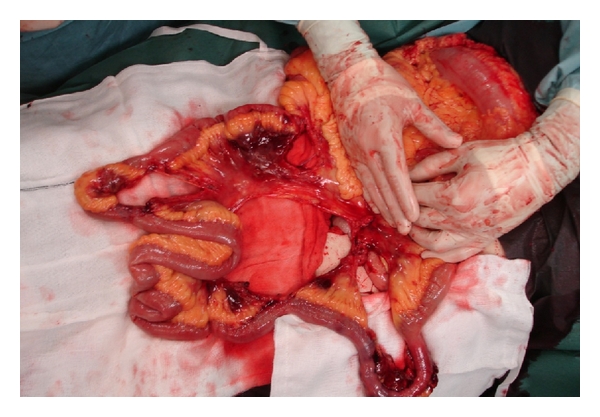
Intra-operative images from Case  1. Small bowel mesenteric shearing injury with devascularisation resulting in small bowel compromise.

**Figure 2:**
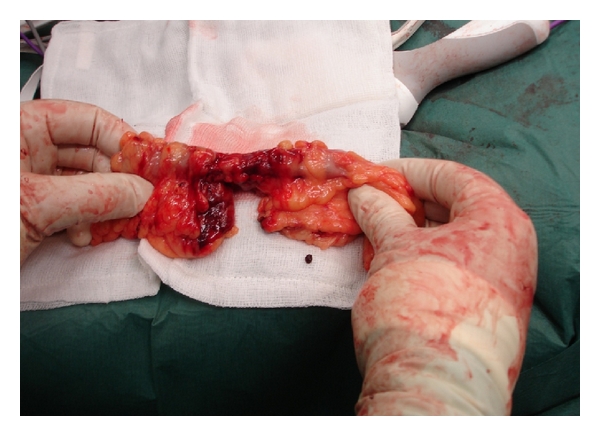
Intra-operative images from Case  1. Small bowel mesenteric shearing injury with devascularisation resulting in small bowel compromise.

All patients were passengers in high impact RTAs; two were front seat passengers and one rear passenger. All three were wearing a three point harness system seatbelt. All patients had a “seatbelt sign” consisting of contusions, petechiae and a band-like pattern of abrasions across the lower abdomen. Two patients had associated orthopaedic injuries, one of which was a lumbar spine injury in keeping with the “seatbelt syndrome” [2]. All patients required emergency laparotomy and bowel resection as a result of mesenteric injury and devascularisation of bowel (Figures 1 and 2). There was no bowel perforation in any of the cases and primary anastomosis was feasible as there was no faecal contamination. All patients made a satisfactory postoperative recovery.

## 3. Discussion

The introduction of seatbelts, and increased compliance with their use [1], has reduced mortality and changed the injury profile associated with RTAs. The reduced mortality is largely attributed to a significant reduction in head injuries with reports of 50% head injury rate in the unbelted population reduced to 32% in the belted population, and a similar reduction in mortality from 7% to 3.2% in high impact RTAs [4]. However, seatbelted occupants in RTAs have been shown to sustain significantly more intra-abdominal injuries, with a two- to three-fold increase in intestinal perforations and mesenteric devascularisation reported [4, 5]. Such injuries occur when a restrained passenger is subject to rapid deceleration. In this series, the intra-abdominal injury sustained by all three passengers resulted in devascularisation of distal small bowel, extending to the caecum in Case  2, and associated with devascularisation of the sigmoid colon in Case  1. None of the patients had a small or large bowel perforation. The mechanism of injury resulting in devascularisation differs from that of perforation. The latter results from compression or crush injury; increased intra-abdominal pressure can cause a closed-loop obstruction at susceptible areas such as the terminal ileum or rectum which leads to perforation [6]. Devascularisation injury is more commonly caused by a combination of compression, crush, and deceleration. Deceleration occurs when the stabilizing portion of an organ ceases forward motion in the torso, while the mobile body part continues to move forward; this can result in shearing injury at fixed points of attachment such as mesentery, resulting in damage and loss of blood supply (Figures 1 and 2). The ileum and jejunum are particularly at risk in this situation via their mesenteric attachment to the posterior abdominal wall, which contains the superior mesenteric vessels. In a seat-belted occupant of a car in collision, when the seatbelt stops the torso suddenly, the small bowel continues to move forward until its mesenteric attachment brings it to a stop, causing shearing of the mesentery, with damage to the superior mesenteric artery (SMA) resulting in small bowel devascularisation—this is the injury observed in all 3 of the cases presented.

Devascularisation is more common in small bowel than in large bowel. In a series reporting 333 cases of blunt abdominal trauma including 31 mesenteric injuries, 30 of these were small bowel devascularisation with only four large bowel devascularisation injuries [7]. Cripps and Cooper reported that small bowel injuries were associated with high velocity impacts irrespective of the degree of abdominal compression, while large bowel injuries occur at both high and low velocity impacts associated with abdominal compression [8]. One of the patients in this series sustained a devascularisation injury to the large bowel (sigmoid colon); notably, this patient also sustained a fracture to the right femur with avulsion of the lesser trochanter and dislocation of a left hip prosthesis indicating that this was likely a very high velocity impact injury. Indeed, the two patients who were front seat passengers sustained multiple injuries including fractures of clavicle, femur, radius, and lumbar spine, while Case  3 who was a backseat passenger had an isolated intra-abdominal injury. This is in keeping with findings that the backseat is a safer environment in the event of a RTA [9]. 

Lumbar spine fractures occur in seatbelt-wearing passengers as a result of hyperflexion of the lumbar vertebrae over an incorrectly applied belt, as in Case  2 who sustained an L1 vertebral fracture. Lumbar spine injuries occur in approximately 5% of RTAs in the seat-belted group and if a lumbar spine fracture is present, the patient is more likely to have an abdominal injury or “seatbelt syndrome” than not (6.2% versus 4.9%) [2, 4]. 

Small bowel injuries such as the cases outlined here can present a diagnostic challenge at initial presentation as signs and symptoms are often delayed. Cases  1 and 3 illustrate this with both being haemodynamically stable at initial presentation with subsequent clinical deterioration, while Case  2 presented with hypotension which responded to initial fluid resuscitation. Frick et al. reported a large series of 5303 cases of abdominal trauma and found that systolic hypotension (SBP < 90 mmHg) was one of the main determinants of morbidity and mortality [10]—it is crucial to recognise and manage these injuries early to avoid adverse outcome. A high index of suspicion should be maintained in all seatbelted occupants of a car involved in a high impact collision. All three patients in this series had significant abdominal contusions in the distribution of the seatbelt. This finding, termed “seatbelt sign”, has been shown to be associated with significant intra-abdominal injury. Sharma et al. [11] reported that patients with a seatbelt sign were twice as likely to have a hollow viscous intra-abdominal injury and three times as likely to have a solid organ injury as those without clinical evidence of a seatbelt sign. Similarly, Chandler et al. [5] report that the clinical finding of a seatbelt sign on presentation greatly increases the likelihood of abdominal injury and the need for operative intervention, in addition to an increased risk of intestinal perforation or mesenteric damage. Of the patients who presented with seatbelt sign, 64% had abdominal injury and 36% needed operative intervention, 21% had small bowel perforation, and 14% had mesenteric injury.

The presence of a seatbelt sign is associated with an increased likelihood of abdominal and intestinal injuries and mandates a heightened index of suspicion

The imaging investigations used in these patients included plain radiography, computed tomography (CT) and focused abdominal sonography in trauma (FAST). In Case  3, the patient became haemodynamically unstable and required emergency laparotomy before any imaging could be performed. 

In cases of small bowel devascularisation injury such as these, there is no single radiological investigation that can provide an accurate and reliable diagnosis. CT is useful and specific in the diagnosis of patients with solid organ injury but lacks sensitivity when diagnosing bowel and mesenteric injury. Breen et al. [12] assessed the diagnostic performance of CT signs in blunt abdominal and mesenteric injury, and found that bowel wall thickening, bowel wall discontinuity, extraluminal air, and mesenteric haematoma are all reasonably specific (84%, 95%, 100%, 94%) but not sensitive (50%, 58%, 44%, 54%). They also reported that the presence of moderate to large volume of intraperitoneal fluid without visible organ damage is an important sign [12]—this was the finding on CT in Case  2. Case  2 was sent for CT only after resuscitation and stabilization of their BP. Not all patients, however, are suitable for CT, particularly if there are concerns regarding haemodynamic stability as seen in Case  3.

FAST scan is a quick and noninvasive investigation that is effective at identifying fluid at different locations in the peritoneum; it is useful in the unstable patient with a suspected intra-abdominal injury. However, it has been reported that if a FAST scan is negative in abdominal trauma, the findings should be confirmed with CT due to lack of sensitivity and the risk of missed intra-abdominal injuries. In a series of 2105 patients, Natarajan et al. reported a high false negative rate of 118, 44 of which required exploratory laparotomy [13]. There is currently no evidence supporting the use of FAST as first line screening in the stable patient [13, 14].

Diagnostic Peritoneal Lavage (DPL) was not employed in these cases, and has become less frequently used as CT imaging technology has improved. However, it can be useful in the patient who is thought to be unsuitable for CT to detect intra-abdominal haemorrhage and guide further management. Furthermore, DPL can be a useful adjunct to CT; comparison studies of DPL ± CT with CT alone as a first line investigation in blunt abdominal trauma have reported that DPL with complementary CT results in low nontherapeutic laparotomy rates and is a sensitive and cost effective approach to the evaluation of blunt abdominal trauma [15–17]

Exploratory laparotomy was indicated in Case  3—the patient became clinically unstable and required immediate intervention, precluding the utilization of any imaging modalities or investigation. This case highlights the importance of relying on clinical signs to guide management, particularly when delay in diagnosis and management significantly increases morbidity and mortality [18]. It has been reported that patients with abdominal injury diagnosed within the first 6 hours had improved postoperative outcomes compared to those whose diagnosis was delayed (median 16 hours). The latter group were more likely to develop complications including sepsis and small bowel obstruction, and had a significantly lengthier hospital stay [4]. Notably in our series, Case  3, the patient who went straight to exploratory laparotomy, had an uncomplicated postoperative course and the shortest length of stay of the three patients. However, other factors including age and additional injuries were likely to have impacted on the postoperative course of the other two patients. 

Early diagnosis and management of intra-abdominal devascularization injuries is critical to optimizing outcomes but of course, attempting to prevent such injuries is hugely important from a public health standpoint. It is recognized that these injuries result from *improper *or* incorrect* seatbelt use. Much of the literature in this regard focuses on the paediatric population, particularly with regard to the use of age-appropriate seat belts [3]. A study which investigated the effects of seating position and appropriate restraint use on the risk of injury to children in motor vehicle crashes showed that children who were restrained inappropriately were at twice the risk of serious injury compared with those who were restrained appropriately; children without restrain were three times at risk of serious injury [19]; the same study confirmed that children in the front seat were at a 40% greater risk of injury. In the adult population, three point harnesses have been shown to be favourable over lap belts and they decrease the risk of “seatbelt syndrome” injuries. In addition to encouraging people to wear their seat belts, public health strategies should also focus on education and awareness of the importance of wearing the seat belt *correctly*. For a seat belt to be worn correctly it should be positioned below the Anterior Superior Iliac Spine (ASIS) and above the femur, and be secure so as to couple the occupant to the vehicle during a crash. Obesity and slouching during long journeys are common ways in which a seat belt can adapt an improper position.

## 4. Conclusion

Intra-abdominal devascularisation injuries sustained via seatbelt use in RTA can be life threatening and diagnostically challenging. The presence of a seatbelt sign should raise the suspicion of a significant intra-abdominal injury. Responding to clinical signs is critical and early diagnosis and management reduces morbidity and mortality. Public health strategies should continue to advocate seatbelt use with an additional focus on the correct manner in which to use a seatbelt in order to reduce the mortality from RTAs.

## Figures and Tables

**Table 1:** Clinical Cases—presentation, investigation and management.

	Case 1	Case 2	Case 3
History	65-year-old female front seat passenger side on collision with truck wearing a seatbelt	60-year-old female front seat passenger head on collision with truck wearing a seatbelt	32-year-old male back seat passenger when head on collision with van wearing a seat belt

Assessment and emergency management	Airway intact	Airway intact	Airway intact
	Breathing uncompromised	Breathing uncompromised	Breathing uncompromised
	BP 105/64 mmHg, HR 64 bpm	BP 84/47 mmHg, HR 60 bpm	BP-109/90, HR 73 bpm
	GCS 15/15	GCS13/15	GCS 15/15
	Positive seatbelt sign—tender lower abdomen, no guarding or rigidity	Positive seatbelt sign—tender with guarding in left upper	Positive seatbelt sign—abdomen initially soft and nontender on examination but progressed to acute rigid abdomen while initial investigations being performed
		BP increased to 118/74 following fluid resuscitation	

Radiological findings	Chest X-ray-fractured left clavicle	FAST- small amount of free fluid around liver and spleen	
	Pelvic X-ray-comminuted fracture of right femoral shaft with avulsion of lesser trochanter and dislocation of left hip prosthesis	CT spine-undisplaced fracture of left lamina of L1 vertebra	Patient became haemodynamically unstable necessitating emergency surgery
	FAST scan-free fluid in right paracolic gutter, pelvis, and around liver	CT abdomen-free fluid around liver and spleen and large haematoma in right abdomen and blood in lesser sac	

Operative detail	Laparotomy findings—1 litre of blood evacuated, extensive small bowel injury, multiple tears in mesentery, devascularisation of 200 cm of distal small bowel and devascularisation of midsigmoid colon with large mesenteric haematoma (Figures 1 and 2)	Laporotomy findings—800 mls of blood in lower abdomen and pelvis, devascularisation injury of terminal ileum and caecum	Laparotomy findings—4.5 Litres of blood in abdomen and pelvis, traumatic devascularisation of the terminal ileum mesentery
	Operative procedure—resection of distal 200 cms of small bowel and caecum with side to side ileocolic anastamosis and Hartmann's procedure	Operative procedure—modified right hemicolectomy with side to side ileocolic anastomosis	Operative procedure—small bowel resection and primary anastomosis

Outcome	Orthopaedic intervention day 10 postop:ORIF right periprosthetic femoral fracture, MUA right distal radius fracture MUA and K-wiring left distal radius fracture, 6 weeks non-weight bearing and physiotherapy	Lengthy postoperative ICU stay complicated by renal failure and sepsis	24 hr ICU admission postoperatively, required transfusion 2 units RBC, uncomplicated postoperative course
	Subsequent stoma reversal	Discharged from hospital day 60 postoperatively	Discharged day 7 postoperatively
